# Latent class analysis suggests four distinct classes of complementary medicine users among women with breast cancer

**DOI:** 10.1186/s12906-015-0937-4

**Published:** 2015-11-19

**Authors:** Garrett Strizich, Marilie D. Gammon, Judith S. Jacobson, Melanie Wall, Page Abrahamson, Patrick T. Bradshaw, Mary Beth Terry, Susan Teitelbaum, Alfred I. Neugut, Heather Greenlee

**Affiliations:** Department of Epidemiology, Mailman School of Public Health, Columbia University, 722 West 168th St, New York, NY USA; Department of Epidemiology, University of North Carolina, 2101 McGavran-Greenberg Hall, CB #7435 Chapel Hill, NC USA; Herbert Irving Comprehensive Cancer Center, Columbia University Medical Center, 161 Fort Washington Ave., New York, NY USA; Department of Biostatistics, Mailman School of Public Health, Columbia University, 722 West 168th St, New York, NY USA; Department of Nutrition, University of North Carolina, 2200 McGavran-Greenberg Hall, CB #7461 Chapel Hill, NC USA; Department of Preventive Medicine, Mount Sinai School of Medicine, 17 E 102nd Street, New York, NY USA; Department of Medicine, College of Physicians and Surgeons, Columbia University, 630 West 168th Street, New York, NY USA; Present address: 722 W. 168th St., Room 733, New York, NY 10032 USA

**Keywords:** Alternative medicine, Breast cancer, Latent class analysis, Epidemiology

## Abstract

**Background:**

Breast cancer patients commonly report using >1 form of complementary and alternative medicine (CAM). However, few studies have attempted to analyze predictors and outcomes of multiple CAM modalities. We sought to group breast cancer patients by clusters of type and intensity of complementary and alternative medicine (CAM) use following diagnosis.

**Methods:**

Detailed CAM use following breast cancer diagnosis was assessed in 2002–2003 among 764 female residents of Long Island, New York diagnosed with breast cancer in 1996–1997. Latent class analysis (LCA) was applied to CAM modalities while taking into account frequency and intensities.

**Results:**

Four distinct latent classes of CAM use emerged: 1) “Low-dose supplement users” (40 %), who used only common nutritional supplements; 2) “Vitamin/mineral supplement users” (39 %), using an abundance of supplements in addition to other practices; 3) “Mind-body medicine users” (12 %), with near-universal use of supplements, mind-body medicine techniques, and massage; and 4) “Multi-modality high-dose users” (9 %), who were highly likely to use nearly all types of CAM. Predictors of membership in classes with substantial CAM use included younger age, more education, higher income, Jewish religion, ideal body mass index, higher fruit and vegetable intake, higher levels of physical activity, receipt of adjuvant chemotherapy, and prior use of oral contraceptives.

**Conclusions:**

LCA identified important subgroups of breast cancer patients characterized by varying degrees of complementary therapy use. Further research should explore the reproducibility of these classes and investigate the association between latent class membership and breast cancer outcomes.

## Background

Complementary and alternative medicine (CAM) is generally defined to include all medical systems, practices and products that are not part of conventional medicine [1]. Individuals in many developed countries use CAM for both illness treatment and disease prevention [2]. CAM use is particularly common among breast cancer patients and survivors; in several samples more than 80 % of subjects reported using CAM [3–5], and many reported using more than one modality [4, 6–8].

Despite high CAM use among breast cancer patients, research of its safety and effects on breast cancer outcomes is lacking, partly due to methodology limitations. Prior observational studies of breast cancer patients have operationalized CAM use as a single dichotomous variable [9], as individual practices or products [4, 5, 8, 10, 11], or employed broad domains of CAM [6, 12, 13] as defined by the National Center for Complementary and Alternative Medicine [1] and others. Using data from the 2002 National Health Interview Survey, Ayers and Kronenfeld conducted a factor analysis of CAM utilization to highlight categories of CAM based on actual use patterns [14]. However, the patterns identified in this population-based sample, in which the prevalence of CAM was relatively low and few subjects used more than one modality [15] may not be applicable to breast cancer survivors.

Although commonly employed in observational research, the analysis of broad CAM domains has been criticized for limiting description of specific products/techniques [13, 16], concealing differences among therapies that could be related to outcomes. Conversely, in studies that analyze the individual effects of a large number of individual practices, chance findings are likely, and teasing out relevant associations may be limited by interactions among modalities [3, 4, 7]. An additional limitation of previous CAM research is the lack of consideration of duration/frequency of treatment, an aspect of CAM behavior that is particularly relevant to causal inference regarding cancer outcomes [17].

Latent class analysis (LCA) is an increasingly popular statistical modeling technique used to uncover heterogeneity in response patterns or clinical characteristics within a population. Its use is common in the social and behavioral sciences and unlike factor analysis, which groups correlated response items, LCA is a person-centered approach [18]. Grouping individuals based on similar patterns of CAM use may address several methodological concerns and provides a practical alternative to traditional subgroup analysis to explore interactions among modalities [19].

Here we present an exploratory latent class analysis of CAM use among breast cancer patients who participated in the Long Island Breast Cancer Study Project [20]. We aim to illustrate the utility of LCA in CAM research, and to explore demographic, clinical and behavioral predictors of different CAM user profiles.

## Methods

### Overview

Data for this study were obtained from the Long Island Breast Cancer Study Project, a federally mandated case–control study to investigate the high incidence of breast cancer in Nassau and Suffolk counties on Long Island, New York [20]. Cases were identified and interviewed shortly after diagnosis in 1996–1997 and, as part of a continuing prospective follow-up study, a second interview among cases was conducted in 2002–2003, which included a questionnaire assessing CAM use. This study was approved by the institutional review boards of Columbia University Medical Center, University of North Carolina, and other collaborating institutions.

### Study population

Female residents of Nassau and Suffolk counties, New York, newly diagnosed with a first primary in situ or invasive breast cancer were identified through a rapid reporting system between August 1996 and July 1997, as previously described [20]. Physician consent was obtained for 1837 cases, 1508 women (82.1 %) completed the baseline questionnaire, and 1414 agreed to subsequent contact. The follow-up interview was conducted with cases or their proxies during 2002 and 2003 [21]. Only data from the 764 patients who personally completed the full follow-up questionnaire were analyzed in this study. Non-responders to the full follow-up questionnaire were on average older, had less education and lower household incomes, were less likely to be non-Hispanic white, more likely to have invasive disease, and less likely to have had a recent mammogram, than responders, as previously reported [10]. In the current sample, 94 % of participants were non-Hispanic and white, and 82 % had invasive (vs in situ) breast cancer [10].

### Data collection

The baseline interview, conducted by trained interviewers roughly three months after diagnosis (mean 96 days), included information on demographics, lifestyle, and breast cancer risk factors. The follow-up interview included questions about the first course of treatment for the first primary breast cancer, and CAM use before and after diagnosis and during treatment. Signed medical records release forms were obtained from case women to abstract data relating to tumor characteristics and treatment during the baseline and follow-up interviews. Informed consent was obtained from all participants before each interview.

### CAM Use assessment

The CAM section of the follow-up questionnaire included detailed questions about 194 modalities in 7 domains that were developed after a review of the literature: 1) vitamin/mineral supplements; 2) herbs/botanicals; 3) non-vitamin/mineral non-herbal over-the-counter (OTC) health products; 4) mind-body medicine techniques; 5) special treatments (including biofeedback, colon cleansing and others); 6) diet change; and 7) practitioner-based therapies. After an affirmative response to ever use of a particular product, participants were asked specifically about the time following diagnosis through the question, “How many total years have you taken this product since diagnosis?” Frequency of using each endorsed modality during treatment for breast cancer was then assessed at the day, week or month level. Although some studies have included prayer as a CAM modality, we excluded it because a high proportion of participants reported using it and because a separate study had shown that prayer fit into a latent construct different from mind-body medicine techniques [14]. We also excluded special treatments such as biofeedback, colon cleansing, and bioelectromagnetic-based therapy due to very low prevalence of use (1.2 %).

We computed a “cumulative dose index” of each CAM therapy by multiplying the reported number of years used since diagnosis by the reported number of times used per week or month. The cumulative dose index value for vitamins/mineral supplements, herbs, and over-the-counter health products was the number of times taken per day multiplied by years taken; the value computed for mind-body practices and CAM practitioners was the number of times used per month multiplied by number of years used; the value for diet was the number of years used since diagnosis.

### Assessment of covariates

Covariates assessed by questionnaire at baseline included age at diagnosis, education, annual household income, marriage history, religion, and race/ethnicity. Known breast cancer risk factors assessed include use of hormone replacement therapy or oral contraceptives, and any first degree family history of breast cancer. Medical history and mammography were also assessed at baseline, in addition to physical activity between menarche and diagnosis, fruit and vegetable intake assessed through a modified Block food frequency questionnaire, life course alcohol use and cigarette smoking history. An unweighted comorbidity index adapted from the Charlson Comorbidity Index [22] was created. An affirmative response at the baseline interview to a history of each of the following conditions contributed 1 point to the index: diabetes, asthma, myocardial infarction, stroke, gallbladder disease, and previous cancer (0, 1, 2 or more). At the follow-up interview, information was collected on weight and height at diagnosis to estimate body mass index (BMI = kg/m^2^), as well as the first course of treatment (chemotherapy/radiation/hormone therapy) for the first primary breast cancer.

Medical records were abstracted at baseline and again at follow-up to ascertain information on tumor staging (in situ vs. invasive), tumor estrogen/progesterone receptor (ER/PR) status, and first course of treatment for the first primary breast cancer. Concordance between the treatment data collected by interview and from medical records was exceptionally high (kappa >90 %) [21], and thus the information assessed by interview is used here.

### Statistical analysis

The analysis included the 23 modalities used since diagnosis at any dose by at least 10 % of women. Modalities used by fewer than 10 % of women were aggregated into six composite variables according to specified CAM domains. Further, in order to increase the precision of the latent class analysis, factor analysis was performed as a data reduction technique prior to conducting the LCA. A two-step process was used for the factor analysis. Because factor analysis of skewed dichotomous data using Pearson correlation coefficients has been shown to underestimate factor loadings [23], we first computed tetrachoric correlations between dichotomous CAM variables [24]. We then conducted the factor analysis on the tetrachoric correlation matrix using principal axis factoring extraction with oblique factor rotation, and determined the number of factors to be retained using the scree test [25]. Modalities that loaded on a single factor with a factor loading of 0.3 or higher on common factors were grouped together. Each CAM grouping generated through factor analysis represented a subset of modalities with correlated use. The cumulative dose of each grouping was calculated by summing the cumulative dose of all modalities making up the respective grouping. Those not loading on any single factor were retained as separate modalities for use in the LCA.

The summed cumulative dose index for each individual modality or CAM grouping was then categorized into three levels with a cutoff at the median dose among those reporting any use: (1) no use since diagnosis; (2) use since diagnosis at a cumulative dose below the median; and (3) use since diagnosis at a cumulative dose above the median. Latent class analysis was performed with SAS PROC LCA [26] using the consolidated 3-level CAM grouping variables. Models specified to contain one through ten latent classes were evaluated based on Akaike’s Information Criterion (AIC), Bayesian Information Criterion (BIC), and the sample-size adjusted BIC. We also considered the reproducibility of the model, as defined by the percentage of iterations yielding the optimal fit, as well as interpretability of the latent classes, when determining the number of latent classes [27].

Using Bayes’ theorem, SAS PROC LCA computes each individual’s posterior probability of membership in each latent class. Subsequent to determining the best fitting number of classes, participants were assigned to classes based on maximum posterior probability. Bivariate associations of demographic, clinical and behavior variables with assigned latent class were examined using Pearson’s chi-squared tests or analysis of variance (ANOVA) for categorical and continuous variables, respectively. Analyses were performed with SAS v.9.3 (SAS Institute Inc., Cary, NC) using two-sided significance tests.

## Results

Between diagnosis and the follow-up interview, study participants reported using CAM practices from a median of 2 domains (range: 0–7) (Table 1). The proportion of women using each individual modality during treatment for their first primary breast cancer has been previously reported [28].

**Table 1:** Prevalence of using multiple domains of complementary and alternative medicine (CAM) since breast cancer diagnosis^a^

Number of CAM domains used^b^	n	Percent
0	38	5.0
1	181	23.7
2	184	24.1
3	138	18.1
4	114	14.9
5 or more	109	14.3

^a^Among Women Diagnosed With Breast Cancer During 1997–1998 Who Completed the Follow-Up Interview in 2002–2003, Long Island, New York

^b^Domains include vitamin/mineral supplements, botanical supplements, other natural products, mind-body techniques, special treatments, dietary change, and practitioner-based complementary and alternative medicine treatments

### Latent class indicators

Preliminary factor analysis identified a 3-factor solution, suggesting that most modalities within domains tended to be correlated in practice. However, Echinacea, green tea, massage, and chiropractic failed to load on any single factor (data not shown). Variables reflecting cumulative dose of each CAM grouping identified, in addition to these 4 individual modalities, were used in the latent class analysis (total of 10 possible modalities). The cumulative dose among those reporting use, as well as the probability of using each modality at a high and low cumulative dose, is reported in Table 2.

**Table 2:** Use prevalence of commonly-used individual CAM modalities and CAM groupings, Long Island, New York (*n* = 764)^a^

	No use	Low dose	High dose	Median (IQR) cumulative dose^b^
Vitamin/mineral supplements^c^	11 %	45 %	45 %	7.7 (2.0–19.0)
Echinacea	86 %	8 %	6 %	0.4 (0.1–0.6)
Green tea	77 %	15 %	8 %	0.6 (0.1–1.3)
Other herbs^d^	79 %	11 %	10 %	1.0 (0.4–4.5)
Natural products^e^	78 %	11 %	11 %	1.0 (0.4–4.6)
Mind-body techniques^f^	59 %	20 %	20 %	19.3 (4.5–135.0)
Diet change^g^	67 %	18 %	15 %	7.0 (4.5–12.0)
Massage	83 %	13 %	4 %	4.5 (2.0–4.5)
Chiropractic	82 %	9 %	9 %	3.3 (1.0–9.0)
Other practitioner-based CAM^h^	88 %	7 %	5 %	2.0 (1.0–3.5)

*Abbreviations*: *CAM* complementary and alternative medicine, *IQR* interquartile range

^a^Indicator variables were determined through factor analysis; CAM groupings represent clusters of individual modalities that tended to be practiced together; cumulative dose for groupings was computed by summing cumulative doses of contributing modalities

^b^Cumulative dose since diagnosis for vitamin/mineral supplements, echinacea, green tea, other herbs, and natural products, expressed as number of times taken per day multiplied by years taken; for mind-body techniques, massage, chiropractic, and practitioner-based CAM, as number of times used per month times number of years taken; and for diet, as the number of combined years since diagnosis

^c^Vitamin/mineral supplements includes all nutritional supplements that include multiple and single vitamins/minerals

^d^Other herbs includes all herbs and botanicals in pill, tea, extract, infusion, oil, powder, or cream form, with exception of echinacea and green tea

^e^Natural product includes all non-herbal, non-vitamin over-the-counter CAM products, predominantly glucosamine, fish oil, coenzyme Q10, flax seed oil, and acidophilus

^f^Mind-body techniques includes support groups; psychotherapy with social worker, psychologist, or psychiatrist; meditation; vizualization/imagery; hypnosis; Reiki, healing touch or other energy therapy; tai chi; qi gong; yoga; dance therapy; art therapy; music therapy; and poetry therapy or journaling

^g^Diet changes considered were vegan/vegetarian; no red meat but do eat chcken and/or fish; organic fruits and vegetables; macrobiotic diet; low-fat diet; high fiber diet; change consumption of soy products; diet or program designed to lose weight

^h^Other practitioner-based CAM includes acupuncture, ayurvedic medicine, traditional Chinese medicine, herbalist, homeopathy, Native American medicine, naturopathic physician, nutritionist/dietician, tibetan medicine, or other practitioner based CAM treatments

### Identification and interpretation of latent classes

The primary goal of model selection was to maximize the number of informative classes while maintaining a balance between parsimony and stability of the models. The 5-class model, with the lowest AIC value, was not sufficiently stable; only 36 % of iterations were associated with the best fitting model. According to the BIC and sample-size adjusted BIC, the 2- and 4-class models, respectively, were preferable (Table 3). After careful examination, we selected the four-class model based on the strong repeatability and interpretability of the model.

**Table 3:** Model fit statistics for estimating classes of CAM users through latent class analysis

Number of classes	Likelihood ratio G2	AIC	BIC	Sample size Adjusted BIC	% of seeds associated with best fit
1	2903.8	2943.8	3036.5	2973.0	100 %
2	2313.5	2395.5	2585.7	2455.5	100 %
3	2202.8	2326.8	2614.4	2417.5	40 %
4^a^	2117.9	2283.9	2668.9	2405.3	100 %
5	2070.4	2278.4	2760.8	2430.6	36 %
6	2028.9	2278.9	2858.7	2461.8	13 %
7	1992.5	2284.5	2961.7	2498.1	3 %

*Abbreviations*: *AIC* Akaike Information Criterion, *BIC* Bayesian Information Criterion, *CAM* complementary and alternative medicine

^a^Selected model contained 4 latent classes

**Fig. 1:**
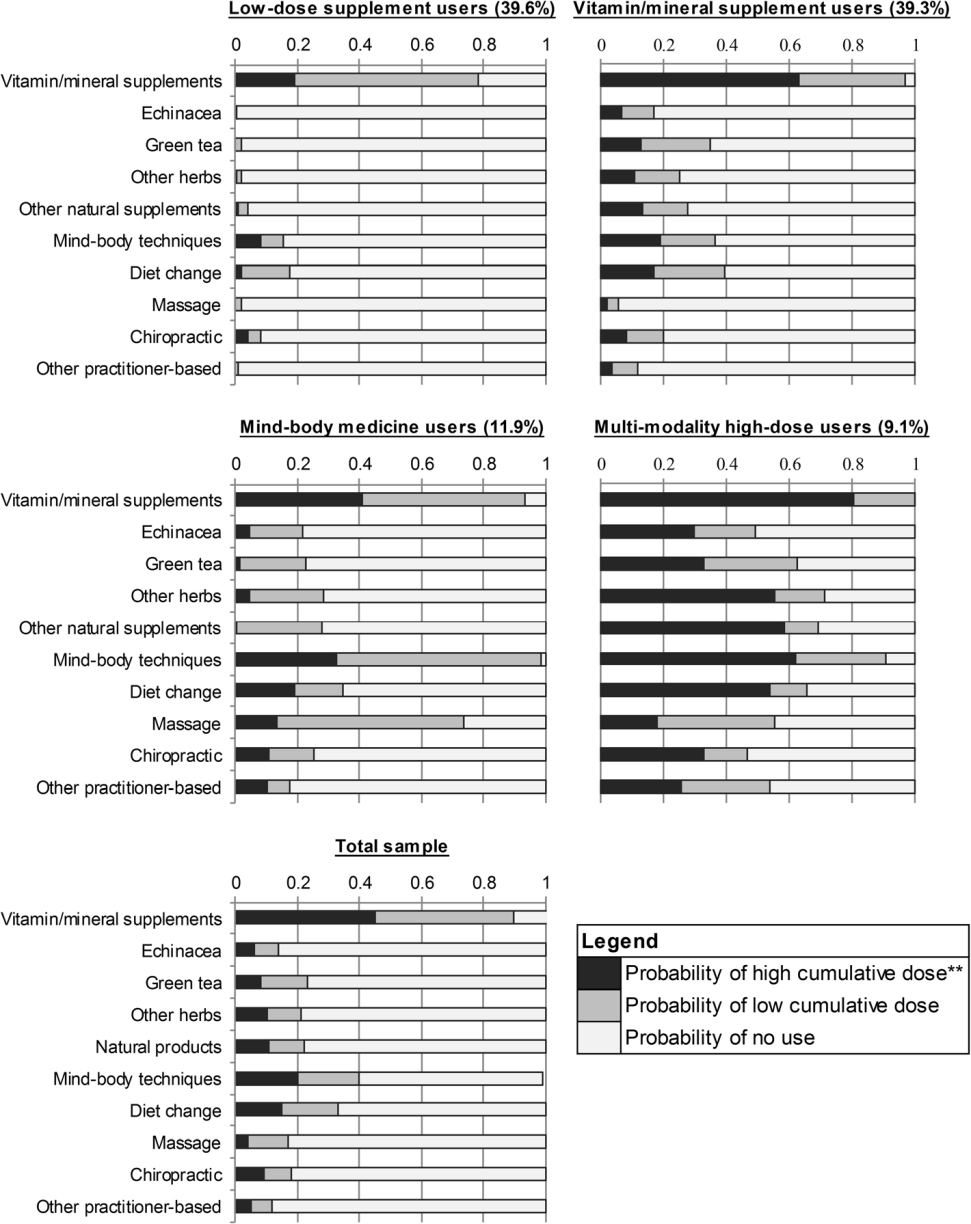
Probability of reporting use of each CAM modality conditional on latent class membership. CAM use assessed in 2002–2003, since a first primary breast cancer diagnosis in 1996–1997, among cases in the Long Island Breast Cancer Study Project, Long Island, New York. Probabilities of class membership identified through latent class analysis; number of classes determined based on model stability, fit statistics, and interpretability of latent classes; high cumulative dose refers to using each modality in excess of the median cumulative dose; abbreviation: CAM, complementary and alternative medicine

The latent class with highest membership probability (39.6 %) was termed “low-dose supplement users” due to their low probability of using all CAM modalities relative to the overall sample. Despite very low CAM use by this class, vitamin/mineral supplement use carried a 59 and 19 % estimated probability of use at a low-dose and high-dose, respectively (Table 4). The second largest class (39.3 %), termed “vitamin/mineral supplement users,” was distinguished by near-universal use of vitamins and minerals, largely in excess of the median cumulative dose, and above average probability of using green tea and diet change. The third largest class, denoted “mind-body medicine users,” (11.9 %) was characterized by near-ubiquitous use of mind-body medicine techniques and massage, predominantly below the median use frequency, and less common use of several other types of CAM. The smallest class, called “multi-modality high-dose users” (9.1 %) was characterized by a relatively high probability of using nearly all CAM modalities. Probabilities of CAM use conditional on latent class membership are shown graphically in Fig. 1.

**Table 4:** Use of CAM modalities since breast cancer diagnosis, conditional on latent class membership^a^

	Low-dose supplement users (39.6 %)		Vitamin/mineral supplement users (39.3 %)		Mind-body medicine users (11.9 %)		Multi-modality high-dose users (9.1 %)	
	Low dose	High dose	Low dose	High dose	Low dose	High dose	Low dose	High dose
Vitamin/mineral supplements	0.59	0.19	0.34	0.63	0.52	0.41	0.19	0.81
Echinacea	0.00	0.00	0.11	0.07	0.17	0.05	0.19	0.30
Green tea	0.02	0.00	0.22	0.13	0.22	0.01	0.30	0.33
Other herbs	0.02	0.00	0.15	0.11	0.24	0.04	0.16	0.55
Natural products	0.03	0.01	0.15	0.13	0.28	0.00	0.11	0.58
Mind-body techniques	0.07	0.08	0.18	0.19	0.66	0.33	0.29	0.62
Diet change	0.16	0.02	0.22	0.17	0.15	0.19	0.12	0.54
Massage	0.02	0.00	0.03	0.02	0.60	0.13	0.37	0.18
Chiropractic	0.04	0.04	0.12	0.08	0.14	0.11	0.13	0.33
Other practitioner-based CAM	0.01	0.00	0.08	0.04	0.08	0.10	0.28	0.26

^a^Probabilities of class membership identified through latent class analysis. Number of classes determined based on model stability, fit statistics, and interpretability of latent classes

Abbreviation: CAM, complementary and alternative medicine

### Predictors of latent class membership

Participants were classified into latent classes corresponding to their highest posterior probability, as derived from the LCA. The mean maximum posterior probability was 0.83, ranging from 0.82 to 0.85 across classes, suggesting low classification error (data not shown). Predictors of membership in the vitamin/mineral supplement users, mind-body medicine users and multi-modality high-doseusers classes were similar, when compared with those of the low-dose supplement users (Table 5 and Table 6). Predictors of membership in classes characterized by substantial CAM use included younger age, more education, higher income, Jewish religion, ideal body mass index, higher intake of fruits and vegetables, higher levels of physical activity, receipt of adjuvant chemotherapy, and prior use of oral contraceptives.

**Table 5:** Demographics and breast cancer risk factors by latent class of CAM use (*n* = 764)^a,b^

	Total sample		Low-dose supplement users		Vitamin/mineral supplement users		Mind-body medicine users		Multi-modality high-dose users	
	*n*	%^e^	*n*	%^e^	*n*	%^e^	*n*	%^e^	*n*	%^e^
Demographics										
Age in years, mean (SD)^d^	56.3	(11.4)	58.8	(12.0)	55.6	(10.5)	50.9	(9.8)	53.1	(10.0)
Age at diagnosis^d^										
< 45 years	136	17.8	48	14.3	51	18.3	24	28.6	13	19.7
45–54 years	231	30.2	84	25.1	88	31.5	34	40.5	25	37.9
55–64 years	204	26.7	85	25.4	81	29.0	18	21.4	20	30.3
65+ years	193	25.3	118	35.2	59	21.2	8	9.5	8	12.1
Religion^c^										
Protestant	173	22.6	82	24.5	65	23.3	13	15.5	13	19.7
Catholic	424	55.5	198	59.1	151	54.1	43	51.2	32	48.5
Jewish	153	20.0	49	14.6	58	20.8	27	32.1	19	28.8
Other/refused	14	1.8	6	1.8	5	1.8	^f^	^f^	^f^	^f^
Annual household income^d^										
Less than $25,000	79	10.3	50	14.9	18	6.5	^f^	^f^	7	10.6
$25,000–$49,999	234	30.6	127	37.9	81	29.0	13	15.5	13	19.7
$50,000–$89,999	266	34.8	100	29.9	94	33.7	44	52.4	28	42.4
Greater than $90,000	185	24.2	58	17.3	86	30.8	23	27.4	18	27.3
Education^d^										
High school graduate or less	301	39.4	185	55.2	92	33.0	10	11.9	14	21.2
Some college	195	25.5	77	23.0	72	25.8	28	33.3	18	27.3
College graduate	111	14.5	31	9.3	56	20.1	16	19.1	8	12.1
Postgraduate	157	20.6	42	12.5	59	21.2	30	35.7	26	39.4
Breast cancer risk factors										
First degree family history of BC										
No	595	77.9	256	76.4	217	77.8	65	77.4	57	86.4
Yes	151	19.8	66	19.7	59	21.2	19	22.6	7	10.6
Prior oral contraceptive use^d^										
Never	376	49.2	190	56.7	135	48.4	25	29.8	26	39.4
Ever	386	50.5	144	43.0	143	51.3	59	70.2	40	60.6
Prior hormone replacement therapy use										
Never	511	66.9	236	70.5	187	67.0	52	61.9	36	54.6
Ever	253	33.1	99	29.6	92	33.0	32	38.1	30	45.5
Menopausal status at diagnosis^d^										
Pre-menopausal	279	36.5	87	26.0	117	41.9	45	53.6	30	45.5
Post-menopausal	465	60.9	239	71.3	158	56.6	38	45.2	30	45.5
BMI at diagnosis^d^										
< 25	375	49.1	139	41.5	158	56.6	36	42.9	42	63.6
25–29	241	31.5	113	33.7	86	30.8	27	32.1	15	22.7
30+	143	18.7	80	23.9	34	12.2	20	23.8	9	13.6

*Abbreviations*: *BC* breast cancer, *BMI* body mass index, *CAM* complementary and alternative medicine, *SD* standard deviation

^a^Participants assigned to class based on highest posterior class membership probability, as determined through SAS PROC LCA

^b^P-values based on Pearson chi-squared tests or analysis of variance (ANOVA)

^c^P < 0.05

^d^P < 0.001

^e^Column percentages may not add up to 100 due to missing data

^f^Values not displayed due to cell sizes < 5

**Table 6:** Health behaviors and clinical characteristics by latent class of CAM use (*n* = 764)^a,b^

	Total sample		Low-dose supplement users		Vitamin/mineral supplement users		Mind-body medicine users		Multi-modality high-dose users	
	*n*	%^e^	*n*	%^e^	*n*	%^e^	*n*	%^e^	*n*	%^e^
Health behaviors										
Mammogram										
No mammogram in last 5 years	32	4.2	19	5.7	8	2.9	^f^	^f^	^f^	^f^
Had mammogram <5 years ago	721	94.4	309	92.2	268	96.1	80	95.2	64	97.0
Cigarette smoking status^c^										
Never	337	44.1	137	40.9	130	46.6	46	54.8	24	36.4
Current	142	18.6	70	20.9	56	20.1	6	7.1	10	15.2
Former	285	37.3	128	38.2	93	33.3	32	38.1	32	48.5
Alcohol use										
Never	258	33.8	120	35.8	86	30.8	32	38.1	20	30.3
Ever	506	66.2	215	64.2	193	69.2	52	61.9	46	69.7
Physical activity since menarche^d^										
None	185	24.2	110	32.8	51	18.3	18	21.4	6	9.1
0–0.69 h/week	172	22.5	77	23.0	57	20.4	23	27.4	15	22.7
0.70–2.6 h/week	184	24.1	81	24.2	70	25.1	13	15.5	20	30.3
≥ 2.7 h/week	181	23.7	52	15.5	81	29.0	28	33.3	20	30.3
Fruit and vegetable intake^d^										
0–34 servings per week	475	62.2	238	71.0	159	57.0	42	50.0	36	54.6
≥ 35 servings per week	281	36.8	95	28.4	116	41.6	40	47.6	30	45.5
Clinical characteristics										
Stage at diagnosis for first primary										
In situ	137	17.9	52	15.5	48	17.2	23	27.4	14	21.2
Invasive	627	82.1	283	84.5	231	82.8	61	72.6	52	78.8
Hormone receptor status for first primary BC										
ER- / PR-	99	13.0	43	12.8	37	13.3	35	41.7	5	7.6
ER- / PR+	27	3.5	11	3.3	14	5.0	14	16.7	^f^	^f^
ER+ / PR-	57	7.5	31	9.3	19	6.8	^f^	^f^	^f^	^f^
ER+ / PR+	292	38.2	135	40.3	101	36.2	32	38.1	24	36.4
First course treatment received for first primary BC										
Chemotherapy^c^	310	40.6	117	34.9	123	44.1	40	47.6	30	45.5
Radiation	464	60.7	206	61.5	180	64.5	42	50.0	36	54.6
Hormonal therapy^c^	462	60.5	207	61.8	177	63.4	39	46.4	39	59.1
Serious comorbidities at diagnosis										
None	463	60.6	194	57.9	180	64.5	49	58.3	40	60.6
One	218	28.5	101	30.2	74	26.5	25	29.8	18	27.3
Two or more	83	10.9	40	11.9	25	9.0	10	11.9	8	12.1

*Abbreviations*: *BC* breast cancer, *BMI* body mass index, *CAM* complementary and alternative medicine

^a^Participants assigned to class based on highest posterior class membership probability, as determined through SAS PROC LCA

^b^P-values based on Pearson chi-squared tests or analysis of variance (ANOVA)

^c^P < 0.05

^d^P < 0.001

^e^Column percentages may not add up to 100 due to missing data

^f^Values not displayed due to cell sizes < 5

## Discussion

We identified four distinct classes of CAM users through a latent class analysis of breast cancer survivors with near-universal use of complementary and alternative medicine. Because much of that use involved vitamin/mineral supplements, we termed as low-dose supplement users patients who were likely to report using common vitamin/mineral supplements but not other CAM since diagnosis. Latent classes were characterized by varying probabilities of using CAM products and techniques in particular groupings.

Results of previous studies suggest that breast cancer patients have a higher probability and more varied CAM use than the general population. While estimates from the 2002 National Health Interview Survey suggest that those in the general population rarely use more than one form of CAM [15], CAM use among breast cancer patients usually involves multiple modalities [4, 6, 7]. Our results are consistent with this observation. Previous studies of breast cancer patients have shown that younger age, higher education and income levels, and receipt of chemotherapy are associated with CAM use in breast cancer patients and survivors [4, 6, 11, 12, 29]. Multi-modality high-dose users and mind-body medicine users are those with a high probability of using modalities generally included in more conservative definitions of CAM use, namely mind-body medicine practices, practitioner-based treatments, and plant-based remedies [29]. Compared with membership in the low-dose supplement users class, membership in the other classes was strongly predicted by known correlates of CAM use, lending credibility to our analytic approach.

To our knowledge only one prior study has assessed CAM use patterns in cancer patients or survivors at the individual level. A cluster analysis conducted by Hok and colleagues in 38 cancer patients characterized CAM users by their therapeutic preferences [7]. Four types of CAM users emerged from their analysis, characterized by the number of NCCAM domains used and a preference for either energy therapies or alternative medical systems and treatment centers. This differs from the present study conducted among a population-based sample of 764 breast cancer survivors in which the most important modalities for discriminating classes were mind-body techniques and massage. This discrepancy is likely explained by differences in the selection of participants, with the small previous study focused on patients with a high commitment to CAM use, with pervasive use of biological-based and mind-body CAM modalities.

Ours is the first study of CAM in breast cancer patients to take into account a measure of relative cumulative CAM dose. Here, a salient aspect of vitamin/mineral supplement users was their tendency to use supplements at a higher intensity/duration than low-dose supplement users. We believe differentiating users based on this factor enhances identification of subgroups of breast cancer patients who are often users of multiple CAM modalities, even though incorporating this strategy weakens precision of parameter estimates. Indeed, data sparseness that resulted from pairing this added complexity of the large number of LCA indicator variables with our relatively small sample size necessitated collapsing CAM modalities prior to performing the LCA [30]. Limited sample sizes in future studies may necessitate aggregating CAM practices using factor analysis as done here, or by combining similar treatment modalities, prior to performing the LCA. Additional strengths of this study include the rigorous consideration of covariates and comprehensive CAM assessment.

Because data on frequency of CAM use were available only during active breast cancer treatment, the cumulative dose component of the analysis was calculated by extrapolating the frequency of use during treatment to all time since diagnosis. Bias therefore may have been introduced regarding modalities correlated with conventional treatments. The findings may also reflect selection bias because non-responders differed from participants on several predictors of CAM use [10]. Other limitations include lack of adjustment for multiple comparisons, although consistency with prior studies lends credibility to the results. It is not clear how these findings may be generalized to different regions and more diverse populations, and whether the use patterns identified here are still relevant to more recently diagnosed breast cancer patients on Long Island.

## Conclusion

In conclusion, this analysis of breast cancer patients suggests four subgroups of women characterized by their use of multiple complementary therapies simultaneously. A large proportion (71.3 %) of study participants reported using two or more forms of CAM following diagnosis and nearly 10 % were committed users of multiple modalities. Latent class analysis was shown to be an effective method for grouping individual breast cancer patients based on multidimensional patterns of CAM use. The generalizability of the latent classes identified here to populations with different socio-demographic and clinical characteristics remains to be determined. However, LCA presents a nuanced approach to data reduction and subgroup analysis in populations with high use of multiple forms of CAM. Further research using LCA-derived classes may be especially useful to investigate causal relationships between CAM and cancer outcomes among patients who use more than one CAM modality.

## Abbreviations

AIC: Akaike’s information criterion
ANOVA: Analysis of variance
BC: Breast cancer
BIC: Bayesian information criterion
BMI: Body mass index
CAM: Complementary and alternative medicine
ER: Estrogen receptor
LCA: Latent class analysis
OTC: Over the counter
PR: Progesterone receptor
SD: Standard deviation
